# Normal reference values of right atrial strain parameters using three-dimensional speckle-tracking echocardiography (results from the MAGYAR-Healthy Study)

**DOI:** 10.1007/s10554-019-01655-0

**Published:** 2019-07-12

**Authors:** Attila Nemes, Árpád Kormányos, Péter Domsik, Anita Kalapos, Nóra Ambrus, Csaba Lengyel, Tamás Forster

**Affiliations:** 1 2nd Department of Medicine and Cardiology Centre, Medical Faculty, Albert Szent-Györgyi Clinical Center, University of Szeged, Semmelweis Street 8, P.O. Box 427, Szeged, 6725 Hungary; 2 1st Department of Medicine, Medical Faculty, Albert Szent-Györgyi Clinical Center, University of Szeged, Szeged, Hungary

**Keywords:** Three-dimensional echocardiography, Right atrium, Strain, Healthy

## Abstract

Classic echocardiographic methodologies offer limited opportunities in assessing right atrial (RA) morphology and function. Three-dimensional (3D) speckle-tracking echocardiography (3DSTE) is a novel imaging method with objective 3D capability in assessing volumetric and functional properties of heart chambers. Normal reference values of different 3DSTE-derived RA strains are not available, therefore the aim of this prospective study was to establish these parameters in healthy subjects. The present study comprised 295 healthy volunteers, from which 110 were excluded due to inadequate image quality. The final population consisted of 185 healthy subjects in the present study (mean age: 32.1 ± 12.2 years, 89 males). Complete two-dimensional echocardiography and 3DSTE have been performed in all cases. While radial strain (RS) does not change significantly over the years in males, in female subjects it increases with age most significantly between at the age of 40–49, and it starts to decline at the age of 50 in females. While females have higher circumferential (CS) and area (AS) strain values, CS and AS decrease with age in both gender. While LS remains almost unchanged in females until ages 40–49 years with a decline above the age of 50, it decreases over the decades in males. 3D strain (3DS) increases with age in both gender, but almost doubles in females in older ages. Specific pattern of strains at atrial contraction could also be demonstrated. 3DSTE-derived RA normal reference values with age-, gender-dependency and regional values are demonstrated in a healthy population.

## Introduction

Right heart failure is a principal cause of morbidity and mortality in different disorders like in pulmonary hypertension [1]. The right atrium (RA) has significant functions during the cardiac cycle: it works as a reservoir in systole, it is a conduit in early diastole and a booster pump in late diastole [2]. Classic echocardiographic methodologies offer limited opportunities in assessing RA morphology and function [2]. Three-dimensional speckle-tracking echocardiography (3DSTE) is a novel imaging method with objective 3D capability in assessing volumetric and functional properties of heart chambers [2, 3]. Strains are objective feature of wall motion and describe wall deformation, which could be calculated from the same 3D model of a particular chamber [2, 3]. In recent studies, usefulness of 3DSTE in assessing RA uni- and multidirectional strains could be demonstrated [4–7]. With these results, different patterns of RA dysfunction could be suggested in several disorders by 3DSTE [8]. However, normal reference values of different 3DSTE-derived RA strains are not available, therefore the aim of this prospective study was to establish these parameters in healthy adult subjects.

## Patients and methods

### Patient population

The present study comprised 295 healthy volunteers, from which 110 subjects were excluded due to inadequate image quality. The final population consisted of 185 healthy subjects in the present study (mean age: 32.1 ± 12.2 years, 89 males). All subjects were taken from the MAGYAR-Healthy Study (Motion Analysis of the heart and Great vessels bY three-dimensionAl speckle-tRacking echocardiography in Healthy subjects), which was organized at our cardiology center to examine normal reference values of 3DSTE-derived parameters among others (‘magyar’ means ‘Hungarian’ in Hungarian language). Subjects were defined healthy if they had no cardiovascular symptoms without use of any medication, no evidence of any disease, normal result of physical examination and routine two-dimensional Doppler echocardiography. All subjects participated in the study on a voluntary basis. All subjects gave informed consent, the study complied with the Declaration of Helsinki and was approved by the institutional human research committee.

### Two-dimensional Doppler echocardiography

Two-dimensional (2D) Doppler echocardiography was used in all cases to exclude any significant morphological or functional cardiac abnormalities and to assess routine data. Complete transthoracic examination has been performed in all subjects extended with 3DSTE. For 2D Doppler measurements Toshiba Artida™ echocardiography machine (Toshiba Medical Systems, Tokyo, Japan) was used equipped with a PST-30SBP (1–5 MHz) phased-array transducer. Left atrial and left ventricular (LV) dimensions and LV ejection fraction were assessed according to recent guidelines. Early (E) and late (A) diastolic transmitral flow velocities were measured by pulsed Doppler echocardiography, while valvular regurgitations were excluded by colour Doppler echocardiography.

### Three-dimensional speckle-tracking echocardiography

**Fig. 1:**
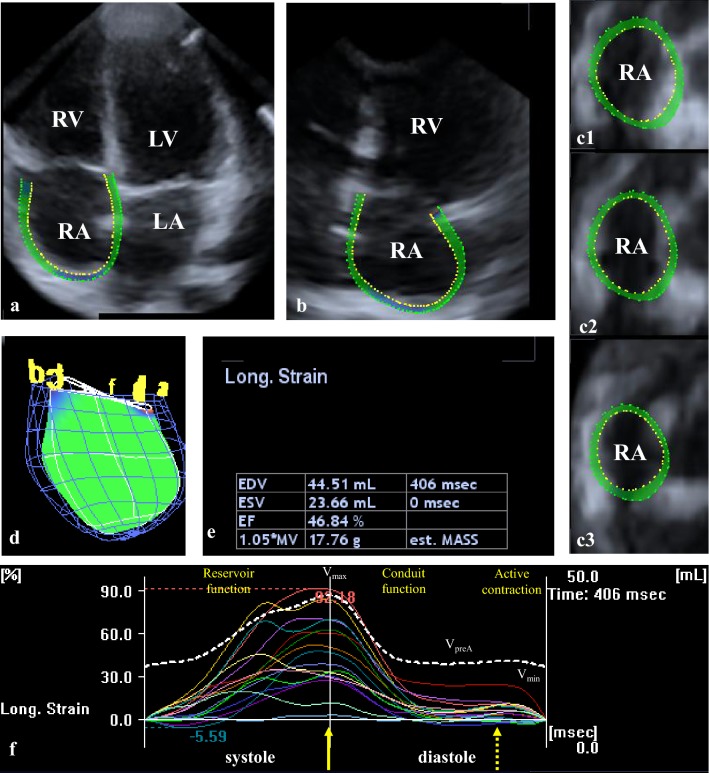
Images from three-dimensional (3D) full-volume dataset showing the right atrium (RA) in a healthy subject are demonstrated (**a**, **b**): **a** apical four-chamber view, **b** apical two-chamber view, **c1** parasternal short-axis view at basal, **c2** mid- and **c3** superior RA levels. In **d** 3D reconstruction of the RA based on 3D speckle tracking echocardiographic analysis is presented. In **e** RA volumetric data are demonstrated. Coloured lines represent segmental RA strains while dashed white line represents RA volume changes over the cardiac cycle (**f**). Yellow arrow represents peak RA strain, while dashed arrow represents RA strain at atrial contraction. *LA* left atrium, *LV* left ventricle, *RA* right atrium, *RV* right ventricle

Apical position was used for data acquisitions with commercially available Toshiba Artida™ echocardiography equipment with a PST-25SX matrix-array transducer (Toshiba Medical Systems, Tokyo, Japan). During 6 cardiac cycles in sinus rhythm (constant RR interval on ECG) and a single breath-hold, R-wave triggered wedge-shaped subvolumes have been acquired from an apical window to create full-volume 3D echocardiographic datasets for later analysis. Offline analysis was performed from acquired 3D datasets using 3D Wall Motion Tracking software version 2.7 (Toshiba Medical Systems, Tokyo, Japan). Automatic analysis included selection of several views including apical four-chamber (AP4CH) and two-chamber views (AP2CH) of the RA and 3 short-axis views at different (basal, midatrial and superior) RA levels at end-diastole. Following optimizations (gain, magnitude etc.), markers were set by the reader to the edges of the tricuspid annulus (TA) and the endocardial side of the superior RA region (Fig. 1). The endocardial surface of the RA was then automatically reconstructed and a 3D RA cast was performed [2, 4–7].

During volumetric measurements, end-systolic maximum RA volume (V_max,_ before mitral valve opening), early-diastolic RA volume before atrial contraction (V_PreA_, at the time of the P-wave on the ECG) and end-diastolic minimum RA volume (V_min_, before mitral valve closure) were assessed for each subject.

During strain measurements, the following parameters were calculated using the same 3D datasets [2, 4–7]

- Unidirectional strains: radial (RS), longitudinal (LS) and circumferential (CS) and complex strains: area (AS) and 3D (3DS) strains.
- For each strains, global (assessing the whole RA) and mean segmental values (average of strains of the 16 segments) were calculated. Segmental RA strain analysis provided superior, midatrial and basal regional RA strain parameters.
- Peak strain (representing RA reservoir phase) and strain at atrial contraction representing booster pump phase were differentiated.

### Statistical analysis

All continuous variables were presented as mean ± standard deviation, while categorical data were demonstrated as frequencies and percentages (%). Comparisons among groups were performed by unpaired Student *t* test and χ^2^ test, when appropriate. A 2-tailed *p* value < 0.05 was considered to indicate statistical significance. All values were considered significantly different at p < 0.05. Statistical analysis was performed by using RStudio (RStudio Team, RStudio: Integrated Development for R. RStudio, Inc., Boston, MA, 2015). For offline data analysis and graph creation, a commercial software package was used (MATLAB 8.6, The MathWorks Inc., Natick, MA, 2015).

## Results

### Demographic and two-dimensional echocardiographic data

A total of 185 healthy subjects were included in the study. This group were further divided into the following categories according to their age: 18–29 years (n = 103; mean age: 23.7 ± 2.8 years, 59 males), 30–39 years (n = 42; mean age: 33.8 ± 2.8 years, 12 males), 40–49 years (n = 15; mean age: 43.9 ± 3.4 years, 7 males) and ≥ 50 years (n = 25, mean age: 57.3 ± 5.7 years, 18 males). Demographic and 2D echocardiographic data were in normal ranges as demonstrated in Table 1.

**Table 1:** Clinical, two-dimensional and volumetric three-dimensional speckle-tracking echocardiographic data of healthy subjects

	Data
n	185
Age (years)	32.1 ± 12.2
Male gender (%)	89 (48)
Weight (kg)	69.5 ± 14.1
Height (cm)	172.2 ± 9.9
Body surface area (kg/m^2^)	1.84 ± 0.24
Two-dimensional echocardiography	
Left atrium (mm)	36.5 ± 3.9
Left ventricular end-diastolic diameter (mm)	47.9 ± 3.7
Left ventricular end-diastolic volume (ml)	105.9 ± 22.9
Left ventricular end-systolic diameter (mm)	33.1 ± 8.7
Left ventricular end-systolic volume (ml)	36.3 ± 9.3
Interventricular septum (mm)	8.9 ± 1.5
Left ventricular posterior wall (mm)	9.0 ± 1.6
E (cm/s)	81.4 ± 17.0
A (cm/s)	63.2 ± 19.0
Left ventricular ejection fraction (%)	65.9 ± 4.6
Three-dimensional speckle-tracking echocardiography	
Maximum right atrial volume (ml)	47.3 ± 14.8
Preatrial contraction right atrial volume (ml)	33.9 ± 11.3
Minimum right atrial volume (ml)	26.3 ± 9.8

### 3DSTE-derived peak RA strains (reservoir function)

**Fig. 2:**
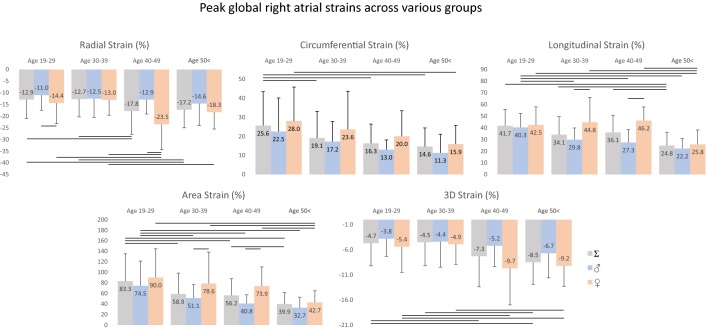
Global peak right atrial strains in males and females over decades. Lines represent significant differences in strains

Peak global RA strains and their gender dependency over decades are demonstrated in Figs. 2 and 4. While RS does not change significantly over the years in males, RS increases with age most significantly between 40–49 years, and it starts to decline at the age of 50 years in females. While females have higher CS and AS values, these parameters decrease with age in both gender. While LS remains almost unchanged in females until 40–49 years and declines above the age of 50 years in females, it decreases over decades in males. 3DS increases with ages in both gender, but almost doubles in females in older ages. Mean segmental LA strains and differences in regional peak RA strains over decades are demonstrated in Tables 2 and 3.

**Table 2:** Age-dependency of three-dimensional speckle-tracking echocardiography-derived peak mean segmental right atrial strain parameters and mean segmental right atrial strain parameters at atrial contraction

	All	Aged 19–29 years (n = 103)	Aged 30–39 years (n = 42)	Aged 40–49 years (n = 15)	Aged ≥ 50 years (n = 25)
Peak mean segmental strains					
RS (%)	− 19.3 ± 7.3	− 19.3 ± 7.1	− 17.3 ± 6.5	− 22.3 ± 8.7	− 21.3 ± 7.3
CS (%)	28.1 ± 15.9	32.0 ± 17.3	24.6 ± 13.7	24.1 ± 11.6	20.2 ± 10.5
LS (%)	41.2 ± 14.5	45.5 ± 13.3	38.0 ± 14.5	40.9 ± 15.3	29.0 ± 10.6*^†‡^
3DS (%)	− 11.5 ± 5.4	− 11.2 ± 5.4	− 10.0 ± 4.8	− 13.7 ± 6.2*^†^	− 13.8 ± 4.9*^†^
AS (%)	78.7 ± 47.8	92.8 ± 52.2	66.5 ± 38.8*	68.4 ± 36.4	47.8 ± 21.8*^†‡^
Mean segmental strains at atrial contraction					
RS (%)	− 7.9 ± 5.0	− 7.3 ± 5.2	− 7.5 ± 4.4	− 11.0 ± 4.3^*^	− 9.0 ± 4.7
CS (%)	12.4 ± 9.0	12.8 ± 9.9	12.6 ± 7.9	12.8 ± 8.9	10.4 ± 6.9
LS (%)	12.1 ± 7.1	12.9 ± 7.7	11.8 ± 6.5	12.3 ± 6.1	9.3 ± 5.4^*^
3DS (%)	− 4.8 ± 4.6	− 4.1 ± 4.9	− 4.7 ± 4.0	− 6.9 ± 3.8*	− 6.6 ± 3.9*^†^
AS (%)	27.3 ± 21.1	30.2 ± 23.9	24.8 ± 17.7	27.6 ± 19.1	19.4 ± 11.3*

*p < 0.05 vs. patients aged 19–29 years

^†^p < 0.05 vs. patients aged 39–39 years

^‡^p < 0.05 vs. patients aged 49–49 years

**Table 3:** Age-dependency of three-dimensional speckle-tracking echocardiography-derived peak regional right atrial strain parameters

	All	Aged 19–29 years	Aged 30–39 years	Aged 40–49 years	Aged ≥ 50 years
RS_basal_ (%)	− 15.4 ± 7.6	− 13.9 ± 7.0	− 14.9 ± 7.1	− 20.6 ± 9.5^†, ‡^	− 19.0 ± 7.0^†, ‡^
RS_midatrial_ (%)	− 19.3 ± 8.5*	− 19.5 ± 8.7^†^	− 16.9 ± 6.3	− 22.5 ± 9.8^‡‡^	− 20.8 ± 9.6^‡‡^
RS_superior_ (%)	− 25.3 ± 14.2*^,^ **	− 27.0 ± 14.9^†, ††^	− 21.4 ± 13.6^‡, ‡‡, †††^	− 24.5 ± 12.6	− 25.7 ± 12.5^&^
CS_basal_ (%)	26.6 ± 12.3	28.3 ± 11.4	23.7 ± 12.1^†^	30.7 ± 14.6	21.8 ± 12.8^†, #^
CS_midatrial_ (%)	24.0 ± 13.2	27.4 ± 14.1	21.2 ± 12.0^††^	20.5 ± 9.9^‡^	17.0 ± 8.3^††^
CS_superior_ (%)	36.3 ± 35.1*^,^ **	44.6 ± 40.7^†, ††^	30.9 ± 25.5^‡‡, †††^	19.3 ± 18.0^†††^	21.9 ± 20.5^†††^
LS_basal_ (%)	44.9 ± 20.3	50.0 ± 18.7	40.0 ± 17.6^†^	48.8 ± 26.1	29.7 ± 18.2^†, ‡, #^
LS_midatrial_ (%)	46.5 ± 18.5	49.4 ± 18.3	45.1 ± 20.1	48.5 ± 16.2	35.7 ± 14.0^††, ‡‡, ##^
LS_superior_ (%)	27.6 ± 21.6*^,^ **	32.8 ± 25.0^†, ††^	24.4 ± 15.7^‡, ‡‡, †††^	17.7 ± 13.9^‡, ‡‡, †††^	17.9 ± 10.1^†††, &, &&^
3DS_basal_ (%)	− 8.7 ± 5.5	− 7.4 ± 4.7	− 8.8 ± 5.4	− 11.7 ± 6.3^†^	− 12.5 ± 6.4^†, ‡^
3DS_midatrial_ (%)	− 10.4 ± 5.6*	− 10.3 ± 5.9^†^	− 8.9 ± 4.1	− 11.9 ± 6.4^‡‡^	− 12.3 ± 5.8^‡‡^
3DS_superior_ (%)	− 17.5 ± 11.5*^,^ **	− 18.6 ± 12.1^†, ††^	− 13.7 ± 10.5^‡, ‡‡, †††^	− 19.4 ± 11.2^‡, ‡‡^	− 18.2 ± 9.9^&, &&^
AS_basal_ (%)	68.6 ± 35.1	77.8 ± 31.1	58.4 ± 31.9^†^	79.9 ± 50.1	41.3 ± 26.7^†, ‡, #^
AS_midatrial_ (%)	79.9 ± 40.8*	90.0 ± 41.0^†^	72.9 ± 44.0^††^	74.1 ± 29.9	53.7 ± 22.6^††, ‡‡, ##^
AS_superior_ (%)	92.2 ± 118.6*	119.7 ± 145.7^†, ††^	69.1 ± 63.9^†††^	42.5 ± 49.5^‡, ‡‡, †††^	48.9 ± 44.3^†††^

*p < 0.05 vs. patients with same strain basal all

**p < 0.05 vs. patients with same strain midatrial all

^†^p < 0.05 vs. patients with same strain basal aged 19–29 years

^††^p < 0.05 vs. patients with same strain midatrial aged 19–29 years

^†††^p < 0.05 vs. patients with same strain superior aged 19–29 years

^‡^p < 0.05 vs. patients with same strain basal aged 30–39 years

^‡‡^p < 0.05 vs. patients with same strain midatrial aged 30–39 years

^‡‡‡^p < 0.05 vs. patients with same strain superior aged 30–39 years

^#^p < 0.05 vs. patients with same strain basal aged 40–49 years

^##^p < 0.05 vs. patients with same strain midatrial aged 40–49 years

^&^p < 0.05 vs. patients with same strain basal aged > 50 years

^&&^p < 0.05 vs. patients with same strain basal aged > 50 years

### 3DSTE-derived RA strains at atrial contraction (booster pump function)

**Fig. 3:**
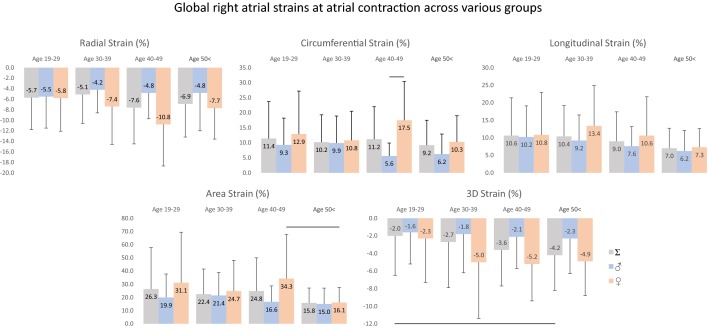
Global right atrial strains at atrial contraction in males and females over decades. Lines represent significant differences in strains

**Fig. 4:**
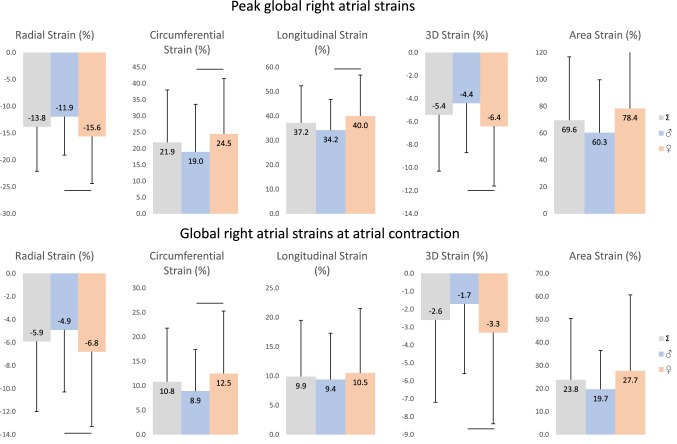
Gender dependency of global peak right atrial strains and global right atrial strains at atrial contraction. Lines represent significant difference in strains between genders

Peak global RA strains and their gender dependency over decades are demonstrated in Figs. 3 and 4. While RS at atrial contraction does not change over decades in males, an obvious increase could be seen in females with the highest value between 40–49 years and a decline over 50 years. A decrease could be seen in CS at atrial contraction in males over decades, it is almost tripled in value between 40–49 years in females. Although LS at atrial contraction is higher in females, it decreases over decades in both genders. AS decreases over decades in males, while females have almost doubled AS values at the ages 40–49 years. 3DS is almost unchanged in males, while doubled but unchanged in older ages in females. Mean segmental LA strains at atrial contraction and differences in regional peak strains over decades are demonstrated in Tables 3 and 4.

**Table 4:** Age-dependency of three-dimensional speckle-tracking echocardiography-derived regional right atrial strain parameters at atrial contraction

	All	Aged 19–29 years	Aged 30–39 years	Aged 40–49 years	Aged ≥ 50 years
RS_basal_ (%)	− 6.3 ± 5.8	− 4.5 ± 4.9	− 7.3 ± 6.3^†^	− 11.4 ± 5.5^†, ‡^	− 9.0 ± 5.7^†^
RS_midatrial_ (%)	− 7.9 ± 5.4*	− 7.7 ± 5.7^†^	− 7.4 ± 4.8	− 11.0 ± 5.4^††, ‡‡^	− 8.1 ± 4.8
RS_superior_ (%)	− 10.1 ± 8.6*^,^ **	− 10.7 ± 9.8^†, ††^	− 8.2 ± 6.2	− 10.3 ± 7.7	− 10.4 ± 7.1
CS_basal_ (%)	10.7 ± 7.2	9.6 ± 6.6	11.5 ± 7.4	14.3 ± 7.8^†^	11.7 ± 8.4
CS_midatrial_ (%)	10.3 ± 7.4	10.1 ± 7.6	10.9 ± 7.3	9.4 ± 8.2	10.5 ± 6.8
CS_superior_ (%)	18.0 ± 20.9*^,^ **	21.7 ± 24.4^†, ††^	17.0 ± 16.1^‡, ‡‡^	11.2 ± 12.6	8.7 ± 9.9^†††, ‡‡‡^
LS_basal_ (%)	11.3 ± 7.8	12.4 ± 8.2	9.5 ± 7.1^†^	13.2 ± 7.4	9.1 ± 6.7
LS_midatrial_ (%)	12.5 ± 8.2	12.8 ± 8.9	12.9 ± 7.8	12.4 ± 6.2	10.9 ± 7.0
LS_superior_ (%)	12.5 ± 13.3	13.9 ± 15.6	12.6 ± 9.3	10.8 ± 12.7	7.2 ± 6.6^†††, ‡‡‡^
3DS_basal_ (%)	− 3.1 ± 5.3	− 1.5 ± 4.7	− 4.0 ± 5.3^†^	− 6.0 ± 5.7†	− 6.1 ± 5.0^†^
3DS_midatrial_ (%)	− 4.5 ± 4.8*	− 4.1 ± 5.1^†^	− 4.1 ± 4.3	− 6.4 ± 3.9	− 5.9 ± 4.1
3DS_superior_ (%)	− 7.8 ± 8.1*^,^ **	− 8.0 ± 9.1^†, ††^	− 6.8 ± 6.3^‡, ‡‡^	− 8.7 ± 7.3	− 8.2 ± 7.2
AS_basal_ (%)	22.0 ± 14.4	23.0 ± 14.6	19.5 ± 14.9	28.3 ± 15.0	18.3 ± 11.1^#^
AS_midatrial_ (%)	24.8 ± 17.5	25.6 ± 18.7	24.3 ± 16.8	27.0 ± 18.0	21.5 ± 13.0
AS_superior_ (%)	38.9 ± 54.3*^,^ **	48.0 ± 66.4^†, ††^	33.6 ± 32.5^‡^	27.4 ± 36.8	18.0 ± 19.4^†††, ‡‡‡^

*p < 0.05 vs. patients with same strain basal all

**p < 0.05 vs. patients with same strain midatrial all

^†^p < 0.05 vs. patients with same strain basal aged 19–29 years

^††^p < 0.05 vs. patients with same strain midatrial aged 19–29 years

^†††^p < 0.05 vs. patients with same strain superior aged 19–29 years

^‡^p < 0.05 vs. patients with same strain basal aged 30–39 years

^‡‡^p < 0.05 vs. patients with same strain midatrial aged 30–39 years

^‡‡‡^p < 0.05 vs. patients with same strain midatrial aged 30–39 years

^#^p < 0.05 vs. patients with same strain basal aged 40–49 years

### Feasibility of 3DSTE measurements

During evaluations, 110 subjects were excluded due to inferior image quality from the total of 295 enrolled subjects, therefore the overall feasibility of 3DSTE-derived RA quantification was 185 out of 295 (63% overall feasibility).

## Discussion

Nowadays, there is an increased attention on the estimation of the right heart due to new opportunities in the treatment of pulmonary circulation. Now there are new possibilities in the non-invasive assessment of RA using recent developments in virtual 3D chamber quantifications during 3DSTE. This imaging methodology is based on a ‘block-matching’ algorithm for echocardiographic imaging. It allows creation of 3D virtual models of certain heart chambers within a relatively short time. By using this 3D cast of atria and ventricles, not only volumetric but also functional parameters can be assessed at the same time [2–7]. RA has a complex function during cardiac cycle including systolic reservoir, early-diastolic conduit and late-diastolic booster pump (active contraction) phasic functions [2]. Functional assessment of the RA involves measurement of volume-based functional parameters and strains respecting the cardiac cycle [2, 3]. Twin-peak time-global/segmental strain curves could be created using the 3D cast featuring RA reservoir (first peak) and RA active contraction (second peak) phases.

RA dysfunction by strain assessments could be demonstrated in several disorders including pulmonary hypertension [9], coronary artery disease [10], heart failure [11], etc. or in top-level athletes [12]. 3DSTE-derived RA strains and their specific patterns [8] could be seen in specific disorders like in corrected tetralogy of Fallot [4], noncompaction cardiomyopathy [5], idiopathic hypereosinophilic syndrome [6] and cardiac amyloidosis [7]. Reference values of RA-LS imaging by 2DSTE were also demonstrated [13]. Moreover, tissue velocity imaging (TVI) and strain rate imaging (SRI) were used to determine normal values in the RA (and LA) in normal subjects. Peak systolic and diastolic velocities of the RA (and LA) decreased from the base to the mid and to the roof, while systolic and diastolic strains and strain rates increased from the base to the mid to the roof [14].

With the present study normal reference values of 3DSTE-derived RA strains have been determined. Specific pattern of changes of different RA strains could be demonstrated over decades with gender differences. The present study could also highlight our attention on the dissimilarities between normal function of the LA and RA in healthy individuals as suggested in an earlier 2DSTE study by Moustafa et al. [15]. In a recent study from our MAGYAR-Healthy Study normal reference values of LA strains have been demonstrated, which showed different behaviour of LA strains as compared to RA strains [16]. RA strains show obvious gender-dependency which could not be confirmed in LA strains. Further studies are warranted to confirm our findings in a larger population and also to further assess RA strains in different pathological states [4–8].

## Limitation section

The following most important limitations should be considered when interpreting the results:

- One of the most important limitation of the study is related to the technical limitation of 3DSTE due to its limited spatial and temporal resolution. It means that average image quality during 3DSTE is worse compared to the corresponding routine 2D echocardiographic image.
- There is a debate whether the atrial septum is part of which atria. In the present study, a 3D virtual RA model was used for assessments including the atrial septum.
- Assessment of atrial strains are only validated against 2D echocardiography [17], 2DSTE [18] and volumetric real-time 3D echocardiography [19].
- Only normal reference values of RA strains were assessed, it was not aimed to determine these values in case of other heart chambers or to measure the volumetric properties of the RA. Strain rate parameters were also not calculated.
- As mentioned in the methods section, twin-peak RA strain curves were analysed featuring RA reservoir (first peak) and atrial booster (second peak) function. Normal reference values for strains characterizing conduit function were not calculated in this study.
- RA highly depends on morphological and functional properties of RV (and pulmonary artery). However, data of all subjects were in normal ranges and had no cardiac or pulmonary diseases or other factors that could affect the results.
- LV segmentation model was used during RA assessments.

## Conclusion

3DSTE-derived RA normal reference values with age-, gender-dependency and regional values are demonstrated in a healthy adult population.
